# Isolation, Molecular Identification and Amino Acid Profiling of Single-Cell-Protein-Producing Phototrophic Bacteria Isolated from Oil-Contaminated Soil Samples

**DOI:** 10.3390/molecules27196265

**Published:** 2022-09-23

**Authors:** Raju Nalvothula, Surekha Challa, Vidyullatha Peddireddy, Ramchander Merugu, M. P. Pratap Rudra, Abed Alataway, Ahmed Z. Dewidar, Hosam O. Elansary

**Affiliations:** 1 Department of Biochemistry, Osmania University, Hyderabad 500007, India; 2 Department of Biochemistry and Bioinformatics, GSS, GITAM, A P., Gandhinagar 530045, India; 3 Department of Nutrition Biology, School of Interdisciplinary & Applied Sciences, Central University of Haryana, Jant-Pali, Mahendergarh 123031, India; 4 Department of Biochemistry, Mahatma Gandhi University, Nalgonda 508254, India; 5 Prince Sultan Bin Abdulaziz International Prize for Water Chair, Prince Sultan Institute for Environmental, Water and Desert Research, King Saud University, Riyadh 11451, Saudi Arabia; 6 Department of Agricultural Engineering, College of Food and Agriculture Sciences, King Saud University, Riyadh 11451, Saudi Arabia; 7 Plant Production Department, College of Food and Agricultural Sciences, King Saud University, P.O. Box 2455, Riyadh 11451, Saudi Arabia; 8 Department of Geography, Environmental Management, and Energy Studies, University of Johannesburg, APK Campus, Johannesburg 2006, South Africa

**Keywords:** phototrophic bacteria, molecular characterization, 16S rRNA gene sequence, amino acid profiling, SEM, HPLC

## Abstract

In the current study, soil samples were gathered from different places where petrol and diesel filling stations were located for isolation of photosynthetic bacteria under anaerobic conditions using the paraffin wax-overlay pour plate method with Biebl and Pfennig’s medium. The three isolated strains were named *Rhodopseudomonas palustris* SMR 001 (Mallapur), *Rhodopseudomonas palustris* NR MPPR (Nacahram) and *Rhodopseudomonas faecalis* N Raju MPPR (Karolbagh). The morphologies of the bacteria were examined with a scanning electron microscope (SEM). The phylogenetic relationship between *R. palustris* strains was examined by means of 16S rRNA gene sequence analysis using NCBI-BLAST search and a phylogenetic tree. The sequenced data for *R. palustris* were deposited with the National Centre for Biotechnology Research (NCBI). The total amino acids produced by the isolated bacteria were determined by HPLC. A total of 14 amino acids and their derivatives were produced by the *R. palustris* SMR 001 strain. Among these, carnosine was found in the highest concentration (8553.2 ng/mL), followed by isoleucine (1818.044 ng/mL) and anserine (109.5 ng/mL), while *R. palustris* NR MPPR was found to produce 12 amino acids. Thirteen amino acids and their derivatives were found to be produced from *R. faecalis* N Raju MPPR, for which the concentration of carnosine (21601.056 ng/mL) was found to be the highest, followed by isoleucine (2032.6 ng/mL) and anserine (227.4 ng/mL). These microbes can be explored for the scaling up of the process, along with biohydrogen and single cell protein production.

## 1. Introduction

Contamination of soil with diesel and petrol leads to pollution. The diesel and petroleum may drift into the lower layers of the soil, which can contaminate the soil environment by blocking the soil pores, hampering the air exchange and decreasing the water-retaining capacity, thus impeding the development of plants and soil organisms [1]. The upper layer poses the risk of the release of aromatic and aliphatic components, which are detrimental to the environment, as these petrochemicals have been proved to possess mutagenic, noxious and carcinogenic properties. Significant quantities of these pollutants can be held in the soil, which might lead to long-lasting pollution of landscape waters, affecting aquatic organisms and, finally, influencing human health via the food-chain link and bioaccumulation [1]. Exposure to volatile organic compounds from petroleum and diesel products might cause inflammation of the digestive or respiratory tract and alterations in the nervous system. Hence, it is essential to remove these products of crude oil, which can lead to persistent toxicity for living organisms. One of the methods by which environmental refurbishment can be achieved is microbial bioremediation, in which microbes use hydrocarbons in the oil products as an energy source [2,3].

The success of bioremediation is associated with the retention of relevant microorganisms that can adapt to various environmental conditions and play an essential role in degrading the pollutants [4,5,6]. Several strains of microorganisms can reduce petroleum hydrocarbons. Some of these bacteria are motile and demonstrate chemotactic feedback, consuming the degraded products of the pollutants as sources of carbon and nitrogen [7]. Mancera-López et al. [8] isolated 37 hydrocarbon-degrading microorganisms, but only 6 strains demonstrated the capability to degrade poly aromatic hydrocarbons (PAHs), aliphatic hydrocarbons (AHs) and total petroleum hydrocarbons (TPHs). These strains were classified as *Pseudomonas pseudoalcaligens*, *Bacillus firmus*, *Bacillus alvei*, *Penicillium funiculosum*, *Aspergillus sydowii* and *Rhizopus* sp., and they were able to eliminate 79%, 80%, 68%, 86%, 81% and 67% of TPHs, respectively [8].

*Penicillium pseudoalcaligenes* and *P*. *funiculosum* eliminated 75% of PAHs, while *Bacillus firmus* and *P*. *funiculosum* degraded 90 and 92% of AHs, respectively. An anonymous strain, AL-12, from a culture of cyanobacteria reduced alkanes (n-C15 to n-C40) found in fresh motor oil. Motor oil in small aliquots was aerobically incubated for up to 50 days, and GC/MSD analysis was performed to measure the hydrocarbon content. Following the incubation for five days, large ratios of the n-alkanes were biodegraded including 70% of n-C15-n-C22, 45% of n-C22-n-C30 and 20% of n-C30-n-C40 [8,9]. Widada et al. [10] classified the diversity of nineteen PAH-degrading bacteria isolated from geographically varied sampling sites in Thailand, Indonesia, Kuwait and Japan. Adenipekun and Oyinkansola [11] reported that *Pleurotustuberregime*, within six months of growth in a contaminated soil sample with 1–40% engine oil concentration, showed an ability to boost nutrient contents in soil. Adenipekun and Isikhuemhen [12] also observed the ability of the white rot fungus *L*. *squadrols* to increase the nutrient composition of engine oil-polluted soil significantly.

Dichlorophenol indophenol (2,6-DCPIP) is classified as an oxidation–reduction indicator that is used in the identification of the process of oxidation of NADH to NAD+ and, hence, is used for isolation of bacteria that degrades hydrocarbon [13]. Koma et al. [14] isolated three kinds of long-chain hydrocarbon-degrading bacteria (*Rhodococcus* sp. NDKK48, *Acinetobacter* sp. ODDK71 and *Gordonia* sp. NDKY76A) that function in the bioremediation of different hydrocarbons. The bacteria NDKK48 and NDKY76A utilize n-alkanes and c-alkanes as their main carbon and energy sources; however, the c-alkane metabolic pathways in these bacteria differ. The strain ODDK71 utilizes a long chain of n-alkanes as a carbon and energy source but does not use c-alkanes. Abioyeet al. [15] described three types of organic waste, including brewery spent grain (BSG), spent mushroom compost (SMC) and banana skin (BS), that proved to be useful for bioremediation in the treatment of soil contaminated with engine oil. The biodegradation of the oil was observed over a period of 84 days. The bacterial counts of the hydrocarbon-utilizing species in the organic wastes were enormous and ranged between 10.2 × 10^6^ and 80.5 × 10^6^ CFU/g [16,17].

Beyond a certain level, diesel contamination might show toxicity to microorganisms and plants [18,19,20]. Phytoremediation, which involves the use of plants, serves as an ecologically and economically viable method for remediating soils polluted with hydrocarbons [21,22]. One of the drawbacks of phytoremediation is that when large numbers of plant species are exposed to pollutants [23], they produce less biomass, have slow growth and require long-term commitment [24,25]. To overcome such problems, plant growth-promoting bacteria [26,27] and contaminant-degrading bacteria could be applied to reduce the toxicity of the pollutants for plants [28,29,30]. Natural petroleum biodegradation processes depend heavily on marine hydrocarbon-degrading bacteria, such as *P*. *lutheri* and *N*. *oculata* [31]. Nine bacterial strains were reported in a contaminated water stream, including *Pseudomonas fluorescens*, *Bacillus* sp., *Alcaligenes* sp., *P*. *aeruginosa*, *Bacillus subtilis*, *Micrococcus roseus*, *Acinetobacter lwoffi*, *Flavobacterium* sp. and *Corynebacterium* sp. [32]. *Candida*, *Yarrowia* and *Pichia*, as well as the fungal taxa Amorphoteca, Neosartorya, Talaromyces and Graphium, were isolated from soil polluted with petroleum and demonstrated to be prospective species for hydrocarbon degradation [32]. *Aspergillus*, *Cephalosporium* and *Pencillium*, a group of terrestrial fungi, were also identified by Singh [33] as possible degraders of crude oil hydrocarbons. Fungi such as *Rhodotorula mucilaginosa*, *Candida lipolytica*, *Geotrichum* sp. and *Trichosporon mucoides* might degrade contaminants such as petroleum compounds [34]. Despite several studies that have shown that unique microbes are useful in biodegradation of soil pollutants [35], many organisms still need to be isolated and evaluated for their potential for the biodegradation of oil- and diesel-contaminated soils. SCPs have been produced using microbial protein synthesis with a variety of natural resources, including grape juice byproduct, date extract and cashew apple juice [36]. SCP manufacturing with cellulose has also been studied [37,38]. The advantages of bacterial SCP manufacture are quick creation times and fast bacterial growth. SCPs can also thrive on other nitrogen sources, such as ammonia, starch, sugars, methane, petroleum fractions, alcohols and sugars [39]. Gaseous hydrocarbons, liquid hydrocarbons and alcohols are the substrates that are used by bacteria. Methanogenic bacteria strains are recommended for the synthesis of SCPs [40]. SCPs are created using a variety of industrial waste products [41]. Microorganisms are now used instead of animal or plant sources, and there have been rapid developments in the current biology of single cell protein (SCP) synthesis procedures. SCP synthesis is an inexpensive and environmentally friendly way for bacteria to use garbage to produce proteins. SCPs are created using a variety of industrial waste items [41]. From synthetic media, *Rhodopseudomonas acidophila* produced 23% SCPs [42]. Forty-five percent SCPs were generated by *Rhodopseudomonas palustris* cultured in simulated wastewater [43]. *Rhodocyclus gelatinosus* grown with cassava waste yielded 56% SCP according to Napavarn [44]. This group of bacteria has not yet been isolated from oil- or petroleum-contaminated soils. Hence, in the present study, the bacteria were isolated from these soils and the SCP production from them is reported.

## 2. Results

Surveying studies were initiated to investigate the effectiveness of the various facets of the growth conditions for the optimization, biomass and biotechnological applications of the selected strains of phototrophic purple non-sulfur bacteria (PNSB) collected from the diesel- and petroleum-contaminated soil samples.

### 2.1. Isolation and Enrichment of the Purple Non-Sulfur Bacteria (PNSB)

PNSB belong to the family *Bradyrhizobiaceae* and are widespread and live freely in nature, such as in water and soil, where enough light is available. PNSB were isolated from the petroleum- and diesel-contaminated soil samples collected from Mallapur, Nacharam, Hyderabad and Karolbagh in Delhi. The collected soil samples were isolated and identified using their culture characteristics (color, shape and size) and nutritional requirements (electron donor, nitrogen sources, growth factors). The pH to be maintained in the enriched medium used—i.e., BP medium—and the peaks for the pigments to confirm the bacterial species in the absorption spectrum analysis were obtained by using the keys from Bergey’s Manual of Systematic Bacteriology. In the current study, the three purple non-sulfur bacteria strains isolated from each soil sample source belonged to the family *Bradyrhizobiaceae* and genus *Rhodopseudomonas*. These three strains were provisionally identified by studying the color of the cell suspension; the morphological characteristics, such as the cell shape and cell size; and the pigmentation characteristics, such as bacteriochlorophylls and carotenoids. These three isolated strains were numbered and given the appropriate names *R. palustris* SMR 001 (Mallapur), *R. palustris* NR MPPR (Nacahram) and *R. faecalis* N Raju MPPR (Karolbagh, Delhi) and then morphologically characterized.

### 2.2. Taxonomic Characterization of the Isolated Bacteria

**Figure 1:**
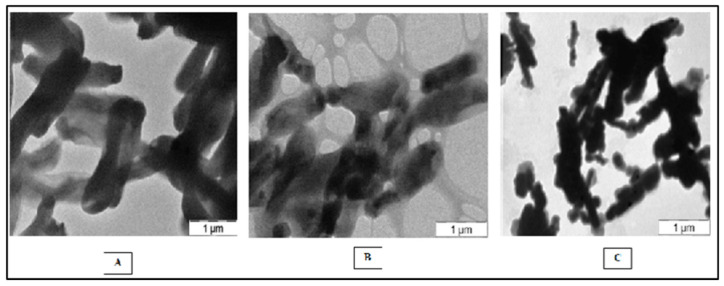
Shapes of the bacterial strains obtained using SEM analysis: (**A**) *R. palustris* NR MPPR, (**B**) *R. palustris* SMR 001, (**C**) *R. feacalis* N Raju MPPR.

The isolated PNSB colonies in the cell suspension were reddish pink in color and the morphologies of the single colonies were observed using a light microscope and SEM. Our observations revealed that the single colonies of the strains SMR001, NR MPPR and N Raju MPPR were rod-shaped bacteria measuring 1 μm (Figure 1A–C).

### 2.3. Absorption Spectrum Analysis

**Figure 2:**
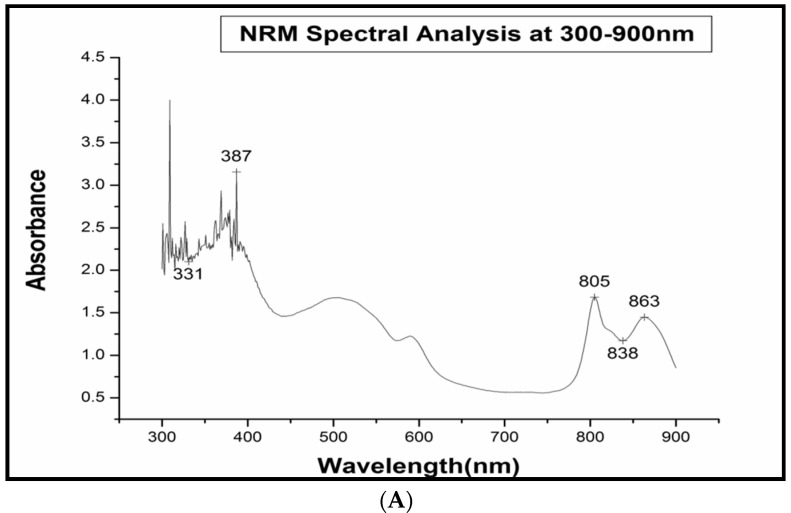

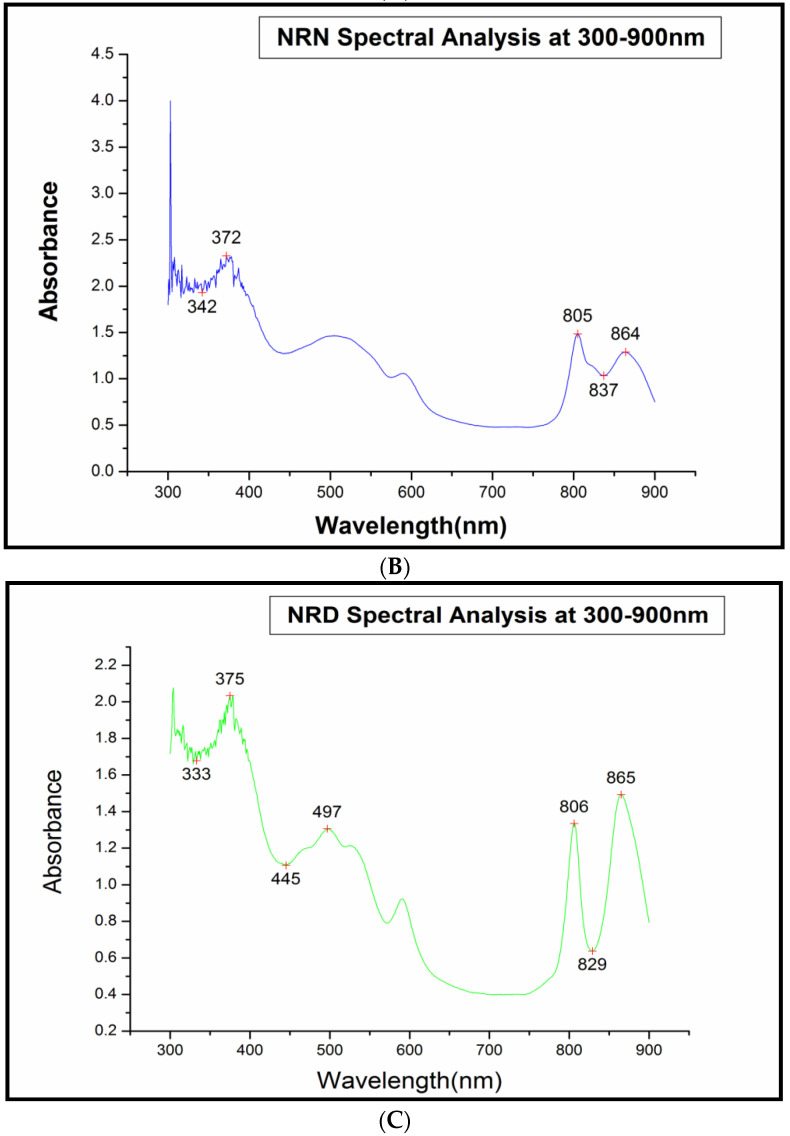
(**A**) Spectral analysis of *R. palustris* SMR 001; (**B**) Spectral analysis of *R. palustris* NR MPPR; (**C**) Spectral analysis of *R. faecalis* N Raju MPPR.

The bacteriochlorophyll and carotenoids present in the three strains SMR001, NR MPPR and N RAJU MPPR were studied using absorption spectrum analysis of the whole cells of the bacteria. The absorption spectra were analyzed in the range from 300–900 nm using a Beckman DU-40 spectrophotometer against a blank solution prepared using 5 g of sucrose dissolved in 3.5 mL of BP medium without the culture or distilled water. The results of the absorption spectrum analysis of the whole cells of the three strains are shown in Figure 2A–C.

As shown in Figure 2A–C, *R. palustris* SMR 001 demonstrated absorption peaks at the wavelengths 387, 805 and 863 nm (Figure 2A), and *R. palustris* NR MPPR demonstrated absorption peaks at the wavelengths 372, 805 and 864 nm (Figure 2C). On the other hand, *R. faecalis* N Raju MPPR demonstrated absorption peaks at the wavelengths 375, 445, 497, 806 and 865. In accordance with the spectral analysis, the bacterial strains isolated from the petroleum- and diesel-contaminated soil samples were characterized and grouped as purple non-sulfur bacteria belonging to the family *Bradyrhizobiaceae*. All the three strains isolated belonged to the genus *Rhodopseudomonas*. However, although the isolated bacteria were identified as the same genus *Rhodopseudomonas*, the species-level identification was undertaken using 16S rRNA gene sequencing and revealed certain variations in the molecular-level genomic analysis.

### 2.4. Genomic Characterization of the Isolated Bacteria

The three bacterial strains collected from contaminated soils around the petroleum and diesel stations at Mallapur, Nacharam (Hyderabad) and Karolbagh (Delhi) were identified and characterized using DNA extraction and purification. The results of this part of the study are given in the Supplementary Materials.

### 2.5. Phylogenetic Relatedness and Sequence Deposition

**Figure 3:**
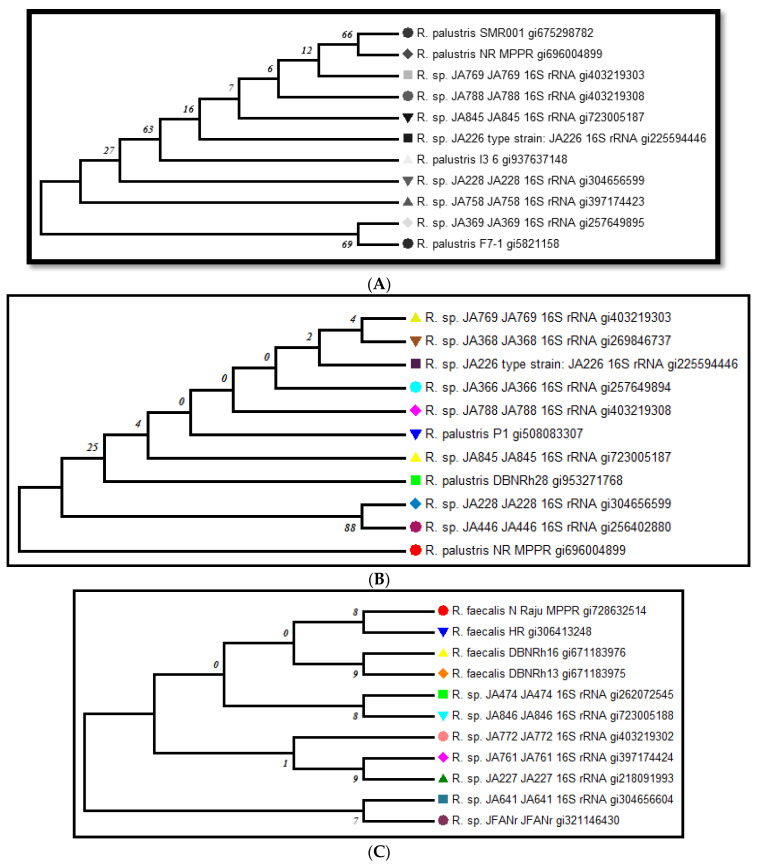
(**A**) Phylogenetic tree (neighbor-joining method) for *R. palustris* strain SMR001; (**B**) Phylogenetic tree (neighbor-joining method) for *R. palustris* NR MPPR; (**C**) Phylogenetic tree (neighbor-joining method) for *R. faecalis* N Raju MPPR.

The phylogenetic relationship between the *R. palustris* strain SMR001 and other PNSB was investigated by means of 16S rRNA gene sequence analysis employing NCBI-BLAST search and a phylogenetic tree. The results of the phylogenetic analyses showed that the isolated strain branched separately but was clustered with the type strains of the species of the genus *Rhodopseudomonas* (Figure 3A). The highest similarities in the sequences for this strain were found with the type strains of *R. palustris* SMR001. These 808 bps sequenced data for the *R. palustris* NRM strain were deposited with the National Centre for Biotechnology Research (NCBI) under the number KJ881378.

The phylogenetic relationship between *R. palustris* NR MPPR and other PNSB was studied by means of 16SrRNA gene sequence analysis employing NCBI-BLAST search and a phylogenetic tree. The results of the phylogenetic analyses showed that the isolated strain branched separately but was clustered with the genus *Rhodopseudomonas* (Figure 3B). The highest similarities in the sequence for this strain were found with respect to the strains of *Rhodopseudomonas* species and *R. palustris*. These 850 bps sequenced data for the *R. palustris* NR MPPR strain were deposited with the National Centre for Biotechnology Research (NCBI) under the number KM 226954.

The phylogenetic relationship between *R. faecalis* N Raju MPPR and other PNSB was examined by means of 16S rRNA gene sequence analysis using NCBI-BLAST search and a phylogenetic tree. The data showed that the isolated strain branched separately but was clustered with the genus *Rhodopseudomonas* (Figure 3C). These 720 bps sequenced data for the *R. faecalis* N Raju MPPR strain were deposited with the National Centre for Biotechnology Research (NCBI) under the number KM 893014.

### 2.6. Physiological and Biochemical Characterization for the Isolated Bacteria

#### 2.6.1. Photo-Organo Heterotrophy

The isolated bacteria were tested for photo-organo heterotrophy by inoculating the culture in sealed sample vials fully filled with BP medium, using succinate/sodium benzoate as the carbon source/electron donor, ammonium chloride as a nitrogen source and cyanocobalamine as a growth factor. The pH of the medium was maintained at 6.8 and the samples were incubated anaerobically under a light intensity of 2000 lux for 6–8 days, at which point they showed reddish-pink colored colonies and cell mass. Culture broth was used for the single colony isolation, employing a paraffin overlay technique to obtain the pure strain, which was used for genomic and characterization studies.

#### 2.6.2. Analysis of Total Amino Acids in the Isolated Bacteria by HPLC

**Figure 4:**
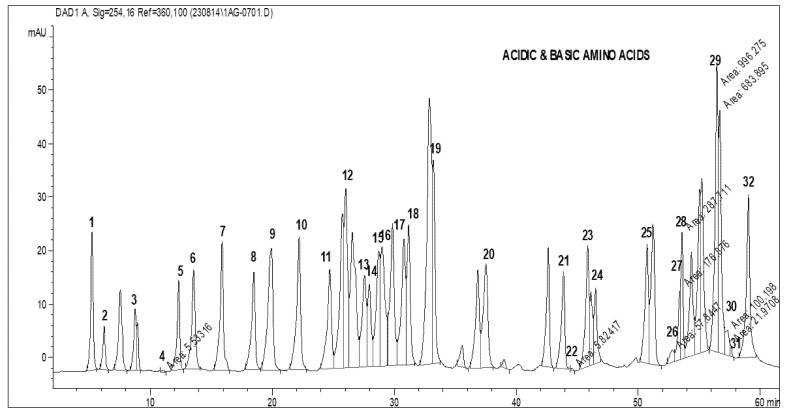
HPLC standard graph showing total amino acids (TAAs) for the estimation of the TAA content present in the isolated strains.

Total amino acid (acidic and basic) analysis was carried out using HPLC (AGILENT). The standard graphs and the sample graphs predicted with the HPLC method are shown in Figure 4 and Table 1.

Table 1 shows the standard values for 32 amino acids. The amino acids were identified based on the peaks observed with standard retention times. The HPLC was run for about 60 min to determine the outflow of all the total amino acids, such as phosphoserine, aspartic acid, glutamic acid, amino adipic acid, oh proline, phosphoenolamine, serine, glycine, asparagine, taurine, threonine, histidine, alanine, β-amino butyric acid, carnosine, proline, arginine, 3-methyl histidine, 1-methyl histidine, anserine, tyrosine, valine, methionine, cystathionine, cysteine, isoleucine, leucine, OH lysine, tryptophan, phenylalanine, ornithine, and lysine. Standards were predicted for the identification of the TAA produced by the isolated bacteria.

**Figure 5:**
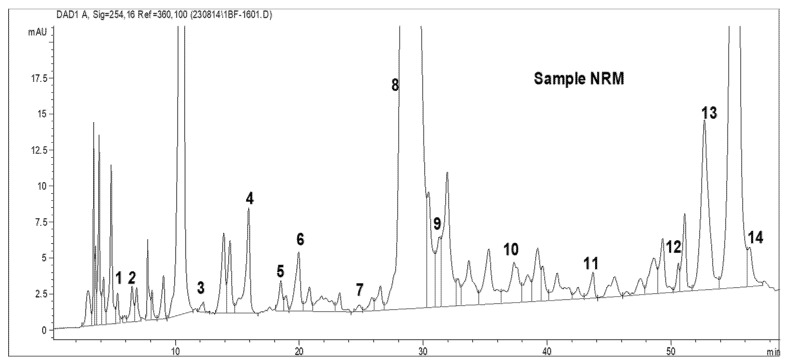
TAA content in *R. palustris* SMR 001 phototrophic bacteria determined by HPLC.

As shown in Table 2 and Figure 5, 14 amino acids in total were produced by *R. palustris* SMR 001. Among the 14 amino acids, carnosine showed the highest concentration, which was about 8553.2 ng/mL, followed by isoleucine (1818.044 ng/mL) and anserine (109.5 ng/mL). Threonine showed the lowest concentration (4.17 ng/mL). Phosphoserine, aspartic acid, hydroxyproline, serine, glycine, asparagine, 3-methyl histidine, tyrosine, cysteine and tryptophan were also found in HPLC analysis.

**Figure 6:**
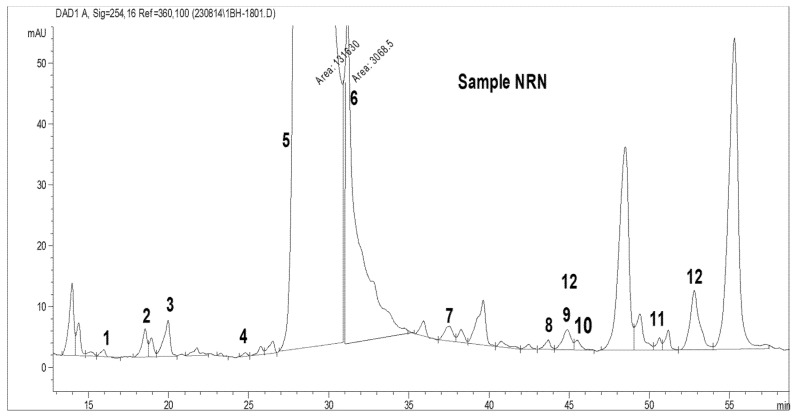
TAA content in the *R. palustris* NR MPPR phototrophic bacteria determined by HPLC.

As shown in Table 3 and Figure 6, 12 amino acids were recorded from *R. palustris* NR MPPR. The highest concentrations, as compared to the standard values for the total amino acids, were recorded, in decreasing order, for carnosine (93,082.9 ng/mL) followed by valine (3877 ng/mL), 3-methyl histidine (1620.5 ng/mL) and isoleucine (1403 ng/mL). Threonine (4.89 ng/mL) and serine (6.764 ng/mL) were observed with the lowest concentrations. Other amino acids present in this bacterium were glycine, asparagine, anserine, tyrosine, methionine and cysteine.

**Figure 7:**
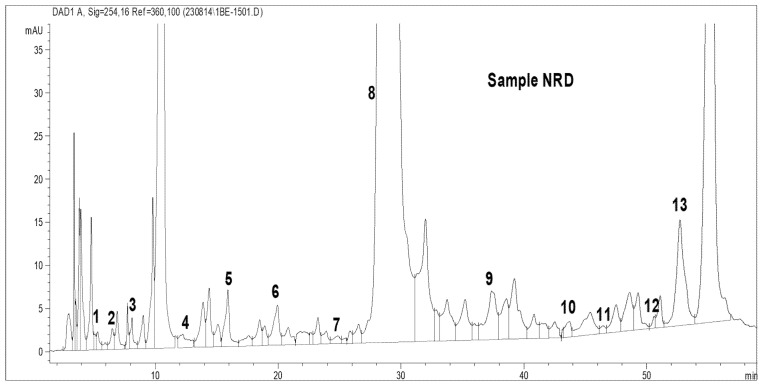
The peaks of the TAA content in *R. faecalis* N Raju MPPR (HPLC).

As shown in Table 4 and Figure 7, 13 amino acids were found in *R. faecalis* N Raju MPPR. The amino acids found in the highest amounts were carnosine, with about 21,601.056 ng/mL, followed by isoleucine (2032.6 ng/mL) and anserine (227.4 ng/mL), which showed values greater than the standard values for the total amino acids analyzed using HPLC. The lowest amounts were recorded for cysteine (9.61 ng/mL) and threonine (14.3 ng/mL). The other amino acids present in this bacterium were phosphoserine, aspartic acid, glutamic acid, hydroxyl proline, serine, asparagine, tyrosine and cystathionine. All other amino acids were recorded as being equal to the standard values for total amino acids estimated with the HPLC method.

## 3. Discussion

PNSB are adaptable organisms that can thrive in a variety of environments [45]. Prototrophs have a high protein composition including essential amino acids and fewer nucleic acids than heterotrophs [46]. PNSB are toxicant-resistant and contain 70–72% crude protein [47]. The essential amino acid makeup of these bacteria is comparable to that of soyabean proteins [44]. Compared to previous similar studies, the methionine and phenylalanine contents in the *Rubrivivax* (Rhodocylcus) *gelatinosus* R1 biomass were low [48]. The amino-acid content of the *R. gelatinosus* R1 biomass was similar to that of algae [49]. PNSB have been suggested by Bender and Phillips [50] as a cheaper alternative protein source for fish feed. PNSB could be used in conjunction with single cell protein (SCP) production according to Imhoff et al. [51]. Kantachote et al. [52] found a protein percentage of 66.7 in wastewater from *Rhodopseudomonas blastica* latex rubber sheet production. Geetha et al. [53] discovered 72–74% SCP protein concentrations in sludge and sago starch processing using *Rhodopseudomonas palustris*, which was employed as aquaculture feed. Elisa et al. [54] found that *Rhodocyclus gelatinosus* cultivated in poultry slaughterhouse effluent, which was used as a feed additive, contained a 67.6% SCP concentration. *Rhodobacter sphaeroides* P47 exhibited 66.6%SCP when grown in a medium dehydrated and obtained from pineapple peel waste production [55]. In *Rhodopseudomonas* sp. CSK01 grown from municipal wastewater, Saejung and Thammaratana [56] reported a 60.1% SCP concentration. Alexandre et al. [57] found a 45% SCP concentration in *Rhodobacter capsulatus* grown in synthetic media. According to Kim and Lee [58], 74%of the SCP from *Rhodopseudomonas palustris* was observed in photosynthetic waste utilized as aquaculture feed. *Brevibacterium* [59], *Bacillus subtilis* [60] and *Methylophilus methylitropous* [61] are a few examples of bacteria that produce significant amounts of SCPs [62]. The best method for addressing the global food crisis is single cell protein (SCP) [63]. Waste management can be improved if waste items are employed as the substrate. For the manufacturing of SCPs, waste such as polysaccharides and hydrocarbons can be employed [64,65].

According to Liao [66], PNSB have been employed for conversion into single cell proteins or hydrogen gas. Due to its high protein content, PNSB biomass is useful for fish feeding [67]. The anaerobic state, light (in lux), pH, carbon, nitrogen, agitation, and growth factors are the variables that can be tuned for PNSB cultivation. In the present study, soil samples were collected from different places where petrol and diesel filling stations are located to isolate the anaerobic conditions of photosynthetic bacteria and characterize them with molecular methods. In total, about 12 to 14 amino acids and their derivatives were identified using HPLC.

## 4. Materials and Methods

### 4.1. Materials

Analytical Grade Chemicals were ssed and purchased from Hi Media, Qualigens and Merck, Mumbai, India. Ultra-Pure Grade (99.9%) nitrogen and argon were used for flushing and to maintain the anaerobic conditions

### 4.2. Area of Study and Sampling Locations

The study area comprised the soil sampling biosphere. Soil samples were taken from Mallapur, Nacharam (located in Hyderabad in Telangana State) and Delhi Karol Bagh diesel and petrol pump stations in India Soil samples were collected from the different places where petrol and diesel filling stations are located for the isolation of photosynthetic bacteria. The soil was collected in air-tight sealed plastic zip-lock bags of 25 g, so that it was possible to collect 15–20 g of soil from the deeper layers at 15 cm depth with the help of spatula. These soil-containing plastic bags were sealed immediately after collection of the soil samples to avoid the entry of aerial microorganisms. The samples were brought to the laboratory within 48 h to avoid changes in the micro-flora present in the soil samples and variations in environmental conditions.

### 4.3. Isolation and Screening of Non-Sulfur Bacteria

Soil sample weighing 1 gm were dissolved in 10 mL of double-distilled water and mixed using vertexing and tenfold dilutions in double-distilled water or Millipore water to make the dilutions reach 10^−9^ for the soil suspensions. These serially diluted samples were sub-cultured into Biebl and Pfennig’s (BP) enrichment medium at a 2000 lux light intensity and at 30 ± 2 °C temperatures for about 6–8 days. Sub-culturing was executed every 60 days. The serially diluted stocks were cultured in enriched Biebl and Pfennig’s 1981 medium to obtain pure strains. These processes were repeated about six times under anaerobic conditions, using the fully sealed tubes for the culturing of the purple non-sulfur bacteria with a paraffin wax-overlay pour plate technique. The composition of the enrichment medium used for the isolation of purple non-sulfur bacteria (PNSB)—i.e., Biebl and Pfennig’s medium, 1981 (mg/liter)—is given in Table 5.

Trace element solution was used in accordance with the above composition (Table 6). Vitamin B_12_ (1 mg/100 mL) was dissolved in double-distilled water and used for the isolation of bacteria as a nutrient in screw-capped bottles, and ferric citrate solution was prepared at concentrations of 0.1%–0.1 mg/100 mL. Both were stored in the refrigerator for further use. The final pH was maintained and adjusted using sterile 1N HCl/1N NaOH.

### 4.4. Maintenance of Stock Cultures

The isolated and enriched stock cultures of purple non-sulfur bacteria were purified through repeated pouring of the resultant medium, consisting of dilutions of 1 mL of inoculum to 20 mL of Biebl and Penning’s (BP) agar medium, onto agar plates at 40–45 °C and allowed to solidify. On the solidified agar, the plates were then layered with molten paraffin wax at 55–60 °C, which solidified upon layering. The plates were rotated mildly by a rounded stirrer while pouring the wax, spreading it uniformly over the agar surface. They were incubated at a temperature of 30 ± 2 °C, with the agar side of the plate exposed to a light intensity of 2000 lux for about 12–15 days, and observed under a microscope after Gram staining. Reddish-pink colored colonies were observed and counted. These cultures were preserved in a refrigerator at 4 °C for further study and sub-cultured every 60 days. The bacteria thus isolated were identified using culture characteristics (including color, size and shape), vitamin requirements, absorption spectra analysis, carbon and nitrogen requirements and bacteriochlorophylls and carotenoids. 

### 4.5. Molecular Identification

The standard protocol given by Ayyaz et al. [67] was used for the isolation of genomic DNA, the amplification of 16S rRNA gene sequencing by PCR, the purification of the PCR product and BLAST analysis.

### 4.6. Scanning Electron Microscopy (SEM)

The morphologies of the bacteria were studied with a scanning electron microscope. The bacterial smears were heat-fixed on a cover slide, which was followed by glutaraldehyde 2.5% treatment for 45 min, dehydration by passing them through 50%, 70% and 90% ethanol. At the final stage, it was treated for 10 min with absolute alcohol each. The gold-coating was applied for the specimens, then scanned and photographed with a scanning electron microscope (Model Hitachi S-530).

### 4.7. Determination of Growth by Optical Density

The growth of the isolated bacterium was determined by using the consistently enriched medium (BP medium) to ensure reliable optical density determination of the bacterial culture broth at 660 nm with a UV-visible spectrophotometer. The enrichment medium without bacterial culture inoculation was used as a blank at room temperature.

#### 4.7.1. Determination of the Biomass of the Bacterial Culture

Bacterial culture biomass was calculated in terms of dry weight in mg/mL. An aliquot of the cultured bacteria was centrifuged at 10,000 rpm for 10 min, and the pellet was washed thrice with distilled water, transferred to a fresh Eppendorf and left for an hour to settle the cell mass. Later, the cell suspension was removed and weighed for the calculation of the wet weight of the bacterial cell mass. The cell mass that was left in Eppendorf was dried at 80 °C overnight and weighed for the dry mass of the bacterial culture at room temperature with a simple balance.

#### 4.7.2. Absorption Spectrum Analysis

The absorption spectrum analysis of the whole cells of the bacteria followed the Trouper and Pfennig method (1981). Five grams of sucrose was added into the 3.5 mL of liquid bacterial broth and dissolved completely for the spectral analysis. The absorption spectrum was analyzed at an absorption ranging from 300 to 900 nm and recorded in a Beckman DU-40 spectrophotometer against a blank liquid, for which the 5 g of sucrose was dissolved in 3.5 mL of medium (BP medium) without the culture or distilled water.

### 4.8. Analysis of Total Amino Acids by HPLC

The analysis of the overall amino acids (acidic and basic) was conducted out using a 1200 series AGILENT reverse-phase HPLC system. The buffers used in the equipment were: buffer A—10 mM sodium acetate, pH 6.4, with 6% acetic acid; and buffer B—buffer A + acetonitrile, 40:60. A flow rate of 1 mL/min and a wavelength detector at 254 nm were used. Then, 100 µg of the sample was obtained then transferred into a clean HPLC vial. Two milliliters of 6 N HCL was added to 50 mL HRB broth tubes, and the HPLC vial was immersed in the tube and the broth tubes were sealed with parafilm. The tube was placed in a dry bath at 60 °C under N_2_ gas for 15 min to maintain inertness, and the temperature was increased to 110 °C and the tubes incubated for 24 h. After 24 h of incubation, 100 µL of 0.1 N HCL was added to the sample and the solution was transferred into a 1.5 mL Eppendorf’s tube, which was vacuum dried at 45 °C for complete dryness. Then, 50 µL of PITC (Edman’s reagent/phenyl isothiocyanate) was added to the pellet and vortexed, and the sample was incubated at 45 °C for 1 h in a thermo-mixer. The sample was vacuum dried, 200 µL of buffer A was added and 20 µL of the sample was loaded into the HPLC for the analysis of the standards and the isolated samples.

## 5. Conclusions

From the current investigation, it can be concluded that purple non-sulfur bacteria are good sources for the production of amino acids and their derivatives and can be explored for the scaling up of the process, as well as biohydrogen production. Since *Rhodopseudomonas palustris* is an adaptable organism and can grow in contaminated soils, it is best-suited for the synthesis of single cell proteins. Future work on SCP production is warranted.

## Figures and Tables

**Table 1:** HPLC standard values of total amino acids (TAAs) for the estimation of the TAA content present in the isolated strains.

Sl. No.	Amino Acid Mixture	Time
1	Phosphoserine	5.235
2	Aspartic acid	6.236
3	Glutamic acid	8.775
4	Amino adipic acid	11.053
5	OH proline	12.341
6	Phosphoenolamine	13.564
7	Serine	15.904
8	Glycine	18.501
9	Asparagine	19.932
10	Taurine	22.210
11	Threonine	24.728
12	Histidine	26.036
13	Alanine	27.574
14	β-amino butyric acid	27.963
15	Carnosine	28.742
16	Proline	29.019
17	Arginine	30.8
18	3-Methyl histidine	31.191
19	1-Methyl histidine	33.217
20	Anserine	37.523
21	Tyrosine	43.884
22	Valine	44.579
23	Methionine	45.868
24	Cystathionine	46.51
25	Cysteine	50.73
26	Isoleucine	52.801
27	Leucine	53.459
28	OH Lysine	53.592
29	Tryptophan	56.451
30	Phenylalanine	57.319
31	Ornithine	57.505
32	Lysine	59.032

**Table 2:** TAA content in the *R. palustris* strain determined by HPLC.

Sl. No.	Amino Acid Mixture	ng/mL of Sample
1	Phosphoserine	21.67761141
2	Aspartic acid	78.5499946
3	OH proline	11.38020833
4	Serine	63.30296313
5	Glycine	12.63515783
6	Asparagine	35.25277984
7	Threonine	4.17106681
8	Carnosine	8553.215786
9	3-Methyl histidine	62.63458849
10	Anserine	109.5590547
11	Tyrosine	35.10529608
12	Cysteine	13.58060892
13	Isoleucine	1818.044983
14	Tryptophan	25.87599117

**Table 3:** TAA content in *R. palustris* NR MPPR (HPLC).

Sl. No.	Amino Acid Mixture	ng/mL of Sample
1	Serine	6.764159039
2	Glycine	31.1292413
3	Asparagine	50.27805319
4	Threonine	4.89298222
5	Carnosine	93082.97738
6	3-Methyl histidine	1620.524745
7	Anserine	64.89758652
8	Tyrosine	30.69745621
9	Valine	3877.198276
10	Methionine	22.1295385
11	Cysteine	16.88288465
12	Isoleucine	1403.788927

**Table 4:** TAA content in *R. faecalis* N Raju MPPR (HPLC).

Sl. No.	Amino Acid Mixture	ng/mL of Sample
1	Phosphoserine	16.47498467
2	Aspartic acid	81.42596694
3	Glutamic acid	81.42878516
4	OH proline	65.06339799
5	Serine	57.93946328
6	Asparagine	47.95234896
7	Threonine	14.39820178
8	Carnosine	21,601.05623
9	Anserine	227.1415528
10	Tyrosine	47.14814429
11	Cystathionine	35.53318299
12	Cysteine	9.617878051
13	Isoleucine	2032.621107

**Table 5:** Composition of enrichment medium (Biebl and Pfennig’s medium).

Chemical/Ingredients Used	Quantity Required (mg) or (mL)
KH_2_PO_4_	500
MgSO_4_·7H_2_O	200
NaCl	400
NH_4_Cl	400
CaCl_2_·2H_2_O	50
Organic carbon	1000
Yeast extract	200
Ferric citrate solution (0.1% *w*/*v*)	5
Trace element solution	1
Cyanocobalamine (1 mg/100 mL)	5

**Table 6:** Trace element composition for the enrichment medium.

ZnCl_2_	70
MnCl_2_·4H_2_O	100
H_3_BO_3_	60
CoCl_2_·6H_2_O	200
NiCl_2_·6H_2_O	20
CuCl_2_·2H_2_O	20
NaMO_4_·2H_2_O	40
HCl	25% (*v*/*v*)-1 mL

## Data Availability

All data obtained during the study are included in this article.

## Supplementary Materials

The supporting information can be downloaded at: https://www.mdpi.com/article/10.3390/molecules27196265/s1.

## Sample Availability

Samples of the compounds are not available from the authors.
