# Supplementary material for: Isolation, Molecular Identification and Amino Acid Profiling of Single-Cell-Protein-Producing Phototrophic Bacteria Isolated from Oil-Contaminated Soil Samples
Source: Molecules. 2022 Sep 23;27(19):6265. doi: 10.3390/molecules27196265 (PMC9572994; doi:10.3390/molecules27196265)
Supplement: Supplementary file 1 [file molecules-27-06265-s001.zip › molecules-1810747-supplementary.pdf]

16s rRNA gene, partial sequence of 945 bp R. palustris strain SMR001

CGGGGGCATACGTCAGTGGCGACGGGTGAGTACGCGTGGGAA
CGTACCTTTTGGTTCGGAACAACACAGGGAAACTTGTGCTATACCGGATAAGCCCTTACGGGGAAAGTTATCGCC
GAAAGATCGGCCCGCGTCTGATTAGCTAGTTGGTGAGGTAATGGCTCACCAGGCGACGATCAGTAGCTGTCTGAG
AGGATGATCAGCCACATTGGGACTGAGACACGGCCAAACTCCTACGGGAGGCAGCAGTGGGGAATATTGGACAA
TGGGCGAAAGCCTGATCCAGCCATGCCGCGTGAGTGATGAAGGCCCTAGGGTTGTAAACTCTTTTGTGCGGGAAG
ATAATGACGGTACCGCAAGAATAAGCCCCGGCTAACTTCGTGCCAGCAGCCGCGGTAATACGAGGGGGCTAGCG
TTGCTCGGAATCACTGGGCGTAAAGGGTGCGTAGGCGGGTTTCTAAGTCAGAGGTGAAAGCCTGGAGCTCAACTC
CAGAACTGCCTTTGATACTGGAAGTCTTGAGTATGGCAGAGGTGAGTGGAACTGCGAGTGTAGAGGTGAAATTC
GTAGATATTCGCAAGAACACCAGTGGCGAAAGCGGCTCACTGGGCCATTACTGACGCTGAGGCACGAAAGCGTG
GGGAGCAAACAGGATTAGATACCCGATAGTCCACGCCGTAACGATGAATGCCAGCCGTTAGTGGTTTACTCACTA
GTGGCGCAGCTAACGCTTTAAGCATTCCGCCTGGGGAGTACGGTCGCAATTAAAACTCAAAGGAATTGACGGGGC
CCGCACAAGC

16s rRNA gene, partial sequence of 850 bp *R. palustris NR MPPR*

TCGGGGCGCATCGTTCAGTGGCAGACGGGTGAGTACGCGTGGGAACGTACCTTTTGGTTCGGAACAACACAGGG
AAACTTGTGCTAATACCGGATAAGCCCTTACGGGGAAAGATTTATCGCCGAAAGATCGGCCCGCGTTGATTAGCT
AGTTGGTGAGGTAATGGCTCACCAAGGCGACGATCAGTAG2CTGGTCTGAGAGGATGATCACACATTGGGACTG
AGACACGGCCCAAACTCCTACGGGGCAGCAGTGGGGAATATTGGACAATGGGCGAAAGCCTGATCCAGCCATGC
CGCGTGAGTGATGAAGGCCCTAGGGTTGTAAAGCTCTTTTGTGCGGGAAGATAATGACGGTACCGCAAGAATAA
GCCCCGGCTAACTTCGTGCCAGCAGCCGCGGTAATACGAAGGGGGCTAGCGTTGCTCGGAATCACTGGGCGTAA
AGGGTGCGTAGGCGGGTTTCTAAGTCAGAGGTGAAAGCCTGGAGCTCAACTCCAGAACTGCCTTTGATACTGGAA
GTCTTGAGTATGGCAGAGGTGAGTGGAACTGCGAGTGTAGAGGTGAAATTCGTAGATATTCGCAAGAACACCAG
TGGCGAAGGCGGCTCACTGGGCCATTACTGACGCTGAGGCACGAAAGCGTGGGGAGCAAACAGGATTAGATACC
CTGGTAGTCCACGCCGTAAACGATGAATGCCAGCCGTTGTGGGTTTACTCACTAGTGGCGCAGCTAACGCTTTAAG
CATTCCGCCTGGGGAGTACGGTCGCAAGATTAAAACTCAAAGGAATTGACGGGGCCCGCACCAGCGGTGGAGCA
TGGTGGTTAATTCGACGCAACGCGCA

16s rRNA gene, partial sequence of 720 bp of *R. faecalis N Raju MPPR*

CCGAAACATTCCTTCATTGGCAGACGGGTGAGTAACGCGTGGGAACGTACCTTTTGGTTCGGAACAACTGAGGGA
AACTTCCTAATACGGATAAGCCCTTACGGGGAAAGATTTATCGCCGAAAGATCGGCCCGCGTCTGATTAGCTAGTT
GGTGGGGTAATGGCCCACCAAGGCGACGATCAGTAGCTGGTCTGAGAGGATGATCACCACATTGGGACTGAGAC
ACGGCCCAAACTCCTACGGGAGGCAGCAGTGGGGAATATTGGACAATGGGCGAAACCCTGATCCAGCCATGCCG
CGTGAGTGATGAAGGCCCTAGGGTTGTAAAGCTCTTTTGTGCGGGAAGATAATGACGGTACCGCAAGAATAAGCC
CCGGCTAACTTCGTGCCAGCAGCCGCGGTAATACGAAGGGGGCTAGCGTTGCTCGGAATCACTGGGCGTAAAGG

GTGCGTAGGCGGGTTTCTAAGTCAGAGGTGAAAGCCTGGAGCTCAACTCCAGAACTGCCTTTGATACTGGAAGTC
TTGAGTATGGCAGAGGTGAGTGGAACTGCGAGTGTAGAGGTGACATTCCTAGATATTCGCAAGATCACCAGTGGC
GAAAGCGGCTCACTGGGCCATTACTGACGCTGAGGCACGAAAGCGTGGGGAGCAAACAGGATTAGATACCCTGG
TAGTCCTCGCCGTCAACGATGAATGCCAGCCGTAGTGGGTTTAC

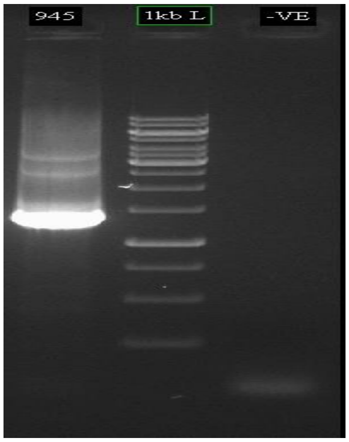

Lane-1	945 (sample)
Lane-2	1kb ladder
Lane-3	negative control

Figure S1. Gel electrophoresis of the elution product of the 16s rRNA gene of the isolated R. palustris SMR 001sample.

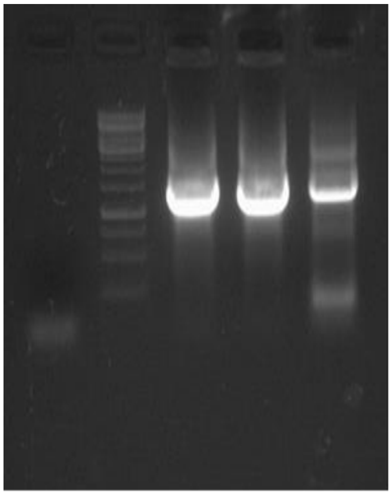

Lane-1	negative control
Lane-2	1kb ladder
Lane-3	893
Lane-4	894
Lane-5	positive control

Figure S2- Gel Electrophoresis figure of the elution product of the 16srRNA gene of the isolated *R. palustris* NR MPPR.

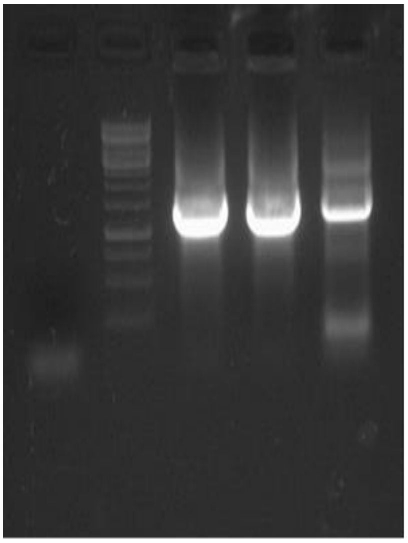

Lane-1	negative control
Lane-2	1kb ladder
Lane-3	893
Lane-4	894
Lane-5	positive control

Figure S3- Gel Electrophoresis figure of the elution product of the 16s rRNA gene of the isolated *R. faecalis N Raju MPPR.*
